# The impact of deregressed foreign breeding values on national beef cattle single-step genomic evaluation

**DOI:** 10.1186/s12711-025-00982-2

**Published:** 2025-07-14

**Authors:** Damilola Adekale, Zengting Liu, Ross Evans, Thierry Pabiou, Reinhard Reents, Dierck Segelke, Jens Tetens

**Affiliations:** 1 Functional Breeding, Georg-August University, Göttingen, Germany; 2 Biometrie, IT Solutions for Animal Production, Verden, Germany; 3 Irish Cattle Breeding Federation, Highfield House, Shinagh, Bandon, Co. Cork Ireland

## Abstract

**Background:**

In recent years, genetic evaluations in cattle breeding have shifted from purely national evaluations to multinational evaluations considering relatives from other countries. Integrating international estimated breeding values (EBVs) into national genomic evaluations presents challenges due to differences in evaluation methodologies and data sources. This study focused on the impact of blending internationally derived EBVs with national EBVs in the single-step genomic evaluation of German beef cattle using three approaches to deregressing EBVs. The national phenotypic data for four breeds (Angus, Charolais, Limousin, and Simmental) were obtained from the routine German beef cattle evaluation of December 2022, and the international EBVs were obtained from the routine Interbeef evaluation of January 2023. Scalar (Garrick (GA), Van Raden (VR)) and matrix deregression approaches were compared for reversibility of EBVs. A forward validation study was used to evaluate the accuracy, dispersion and level bias obtained in a purely national single-step evaluation, and single-step genomic evaluations blended with DRPs obtained from the three deregression approaches.

**Results:**

A validation study based on forward prediction showed improved accuracy, and reduced dispersion bias in the EBVs blended with international EBVs compared to purely national EBVs, particularly for the direct and maternal effects of 200-day weight. As expected, Pearson correlation analysis revealed that the matrix deregression (> 0.99) approach outperformed the scalar deregression approaches (0.75–0.99), exhibiting a greater correlation between the EBVs obtained from DRPs and the EBVs obtained from phenotypes across the various breeds and traits in our study. A forward validation study with and without integrating foreign data across the three deregression methods showed improvement in reducing dispersion bias, as indicated by the regression coefficient. The GEBVs from an evaluation incorporating foreign information with national data showed a higher correlation to the GEBVs from a truncated evaluation than those from an evaluation without foreign information.

**Conclusions:**

These findings underscore the importance of accurately integrating foreign EBVs to enhance national genomic evaluations and genetic progress in livestock populations. Our results show that the matrix approach to deregressing EBVs performs optimally across traits and breeds. However, the VR deregression approach can serve as an alternative in situations where the matrix deregression approach might be too technical to implement.

## Background

The Interbeef evaluation [1] enables breeders to obtain more choices of animals to mate with and increases the associated reliability of estimated breeding values (EBVs). The Interbeef evaluation is a pedigree-based genetic evaluation system providing internationally EBVs on each country's scale for all animals in the country’s pedigree and sires that meet the criteria for international publishing [2]. The Interbeef evaluation is based on a single-trait, multiple-country pedigree-based model. In general, the reliability of EBVs increases when the information of relatives of animals recorded in other countries is considered as in the Interbeef international evaluation [3, 4]. Since 2023, the breeding value evaluation for beef cattle in Germany has applied a multi-trait single-step SNPBLUP model involving birthweight (BW), 200-day weight (200-DW), 200-day muscling (200-DM), 365-day weight (365-DW), and 365-day muscling (365-DM) [5]. The differences in approaches and data sources used in the national and international evaluations can lead to differences in EBVs and animal rankings obtained from the Interbeef and national evaluations. Due to the utilization of distinct sources of information at national and international evaluations, opting for either the internationally EBVs (EBV_INT_) or the genomically enhanced EBVs estimated from only national data (GEBV_NAT_) [5] may lead to the loss of valuable information related to the discarded EBVs. The differences in evaluation methods—such as Interbeef pedigree-based versus national genomic evaluations, the traits considered, and the number of animals included in the national and Interbeef evaluations—result in inconsistencies between the EBVs of closely related individuals excluded from the international evaluation and those of national bulls that were not incorporated into the international evaluations [6, 7]. In the German case, only animals with a 200-DW (analogous to Adjusted Weaning Weight (AWW) in the Interbeef evaluations) record and their pedigree are subjected to the Interbeef evaluations. However, animals measured for any of the five national phenotypes (BW, 200-DW, 200-DM, 365-DW, 365-DM) and their pedigrees are included in the national evaluation. Furthermore, there is genuine interest in combining both EBVs into a single measure of genetic merit for practical breeding purposes.

Integrating international EBVs in the national EBV estimation is known as '*blending*' [6]. This process involves the combination of EBVs estimated from multiple but overlapping sources of information. It is important to note that this blending differs from the blending of the genomic (*G*) and pedigree based (*A*) relationship matrices in single-step GBLUP evaluations. Optimally, blending approaches should enable the unbiased dissemination of foreign information to every animal in the national evaluation—including those excluded from the international evaluation—through relationships derived from the national pedigree. Several techniques have been developed to integrate international EBVs into a national evaluation to avoid the data loss associated with choosing one or the other [8]. Widely adopted approaches for blending two EBVs derived from overlapping sources of information combine foreign information with the national EBVs by converting EBVs and associated reliabilities to pseudo records such as deregressed proofs (DRPs) and effective record contributions (ERCs). During the blending of EBVs obtained from overlapping sources of information, it is critical to prevent the double counting of national phenotypic information. The EBV of an animal is a combination of the information from its own records, records of relatives, and progeny through the relationship matrix [7, 9]. Subsequent analyses that use DRPs and ERCs often assume that these pseudo-phenotypes derived from the EBVs are measured on the animal itself. Consequently, appropriate deregression approaches should correct the EBVs for information from relatives to prevent the double-counting of information in downstream analyses of DRPs [8].

In beef cattle, several traits are maternally influenced and thus genetically modelled, so the trait is a sum of additive direct and maternal genetic effects. The maternal effect is defined as the additive genetic ability of the dam to provide a suitable environment [9, 10]. The maternal breeding value can include a variety of factors, such as the dam's ability to provide sufficient nutrition during pregnancy, suckling and her behavioural characteristics that influence the observed phenotype in the offspring. Considering that the maternal breeding value is not a trait that is directly measurable, additional considerations are required when blending foreign and national EBVs.

To our knowledge, there has been no consensus on the frameworks for integrating direct and maternal EBV_INT_ into national evaluations for beef cattle populations. Studies [3, 11] have shown approaches to integrate foreign EBVs into national evaluations of beef cattle. The approach implemented by Pabiou [11] is the current approach used to blend international EBVs into the Irish national evaluation. Bonifazi [3] blended EBV_INT_ with EBV_NAT_ by deregressing EBV_INT_ and EBV_NAT_ using a scalar deregression approach [12] in the Italian Limousin population. However, literature [13] has reported that the scalar deregression approach causes the variance of EBVs in downstream genetic analyses to be biased. In the national evaluations of dairy cattle in several countries and the official MACE evaluation, the matrix deregression [13, 14] is implemented in many blending frameworks [15–17].

In this study, we aimed to validate deregression approaches to integrate internationally derived pedigree-based EBVs into the national single-step beef cattle evaluations. Mainly, this study focused on:

1. Comparing scalar [12, 18] and matrix deregression [13, 14] approaches using the direct and maternal genetic effects from national and international evaluations.
2. Validating the DRPs obtained from scalar and matrix deregression approaches with a forward validation method by cutting off the last three birth years in a truncated evaluation. The 200-day weight, analogous to the Interbeef trait AWW, was used as a case study to validate the integration of foreign EBVs.

## Methods

### Model

The official Interbeef breeding value estimation is based on a pedigree-based animal model accounting for across-country interactions [1, 3]. The Interbeef model is a single-trait BLUP animal model with direct and maternal genetic effects in which each country's data is modelled as a different but correlated trait [1]. The 200-DW phenotype is measured between 90 and 280 days of life. A linear adjustment for the frame and age of the animal at measurement creates the Interbeef AWW phenotype. The genetic merit in German beef cattle populations is evaluated using a multi-trait single-step SNPBLUP model [5]. To integrate the Interbeef EBVs with the national EBVs, we obtained direct and maternal EBVs from a reduced national pedigree-based model containing only the phenotypes of German animals sent for the Interbeef evaluation. On the national level, a single-trait BLUP animal model with maternal effects was implemented to align the national model with the Interbeef model.

### Integration of Interbeef results into the national evaluation

Similar to [3], our approach to integrating direct and maternal Interbeef EBVs and reliabilities (RELs) from the Interbeef evaluation into the national single-step genetic evaluation consists of five steps.

* Conventional PBLUP national evaluation*: EBVs and associated reliabilities were obtained for direct (EBV_NAT_dir_, REL_NAT_dir_) and maternal genetic effects (EBV_NAT_mat_, REL_NAT_mat_). The EBVs were estimated using a single-trait pedigree BLUP animal model with maternal effects from the phenotypic data of animals with a 200-DW record and the national pedigree.
  1 $$\mathbf{y}=\mathbf{X}\mathbf{b} + { \mathbf{Z}}_{1}\mathbf{h}+ {{ \mathbf{Z}}_{2}\mathbf{a}+ \mathbf{Z}}_{3}\mathbf{m}+\mathbf{e}$$
  where $\mathbf{y}$ is the vector of 200-DW records, $\mathbf{b}$ is a vector of fixed effects, $\mathbf{X}$ is the design matrix linking the fixed effects to the records, $\mathbf{h}\sim N\left(0,{\mathbf{I}\upsigma }_{\text{h}}^{2}\right)$, $\mathbf{a}\sim N\left(0,{\mathbf{A}\upsigma }_{\text{a}}^{2}\right)$, and $\mathbf{m}\sim N\left(0,{\mathbf{A}\upsigma }_{\text{m}}^{2}\right)$, $\mathbf{e}\sim N\left(0,{\mathbf{I}\upsigma }_{\text{e}}^{2}\right)$ are vectors of random herd-year effects, random animal effects, maternal effects and residual effects, respectively. ${\mathbf{Z}}_{1}$, ${\mathbf{Z}}_{2}$ and ${\mathbf{Z}}_{3}$ are design matrices linking the random effects to the records. The single-trait two effect model involved a genetic correlation between the direct and maternal effects of − 0.3. The reliabilities using national data were estimated for the direct and maternal effects. The corresponding international EBVs (EBV_INT_dir_, EBV_INT_mat_) and associated reliability (REL_INT_dir_, REL_INT_mat_), including German animals, were obtained from the Interbeef evaluation.
* Obtaining Effective Record Contributions (ERCs)*: Using the reliabilities obtained in step 1, the effective record contributions for direct (ERC_NAT_dir_, ERC_INT_dir_) and maternal genetic effects (ERC_INT_mat_, ERC_INT_mat_) were independently obtained using the single-trait ERC from the reversed reliability approximation algorithm [19]. The reverse reliabilities were estimated as uncorrelated between the direct and maternal effects.
*Deregression*: Using the ERC obtained in step 2, EBV_NAT_ was deregressed using the matrix [13, 14] and scalar (Garrick: DRP_GA_ and Van Raden: DRP_VR_) [12, 18, 20] approaches to obtain DRP_NAT_dir_, DRP_INT_dir_, DRP_NAT_mat_, and DRP_INT_mat_. The matrix deregression procedure involves setting up the mixed model equations (MME) for a pedigree-based model that includes all animals under consideration. The procedure for implementing matrix deregression is detailed in [13]. Following [12], DRP_GA_ was computed by
  2 $${DRP}_{k}={PA}_{k}+ \frac{\left({EBV}_{k}- {PA}_{k}\right)}{{REL}_{k\left(o+p\right)}},$$
  where $${PA}_{k}$$ is the parent EBV average of the individual $$k$$ computed as $$\frac{{EBV}_{sire}+{EBV}_{dam}}{2}$$, $${REL}_{k\left(o+p\right)}$$ is the reliability due to the individual's performance and of its progeny computed as $$\frac{{dERC}_{k}}{{dERC}_{k}+ \lambda }$$, $$\lambda$$ is defined as $${\raise0.7ex\hbox{${\sigma_{e}^{2} }$} \!\mathord{\left/ {\vphantom {{\sigma_{e}^{2} } {\sigma_{a}^{2} }}}\right.\kern-0pt} \!\lower0.7ex\hbox{${\sigma_{a}^{2} }$}}$$, and $${dERC}_{k}$$ is the individual deregressed ERC. Following a third approach [18], DRP_VR_ was calculated as
  3 $${DRP}_{k}={PA}_{k}+\left({EBV}_{k}-{PA}_{k}\right)\left[\frac{{ERC}_{k}}{{dERC}_{k}}\right],$$
  where ${ERC}_{k}$ is the ERC associated with ${EBV}_{k}$, and ${dERC}_{k}$ is the deregressed ERC for the animal $k$.
*DRP Blending*: To avoid double counting DRPs, the following formulae were used for blending [8, 21].
  4 $${ERC}_{\left(dir,mat\right)}^{*}= {ERC}_{INT} - {ERC}_{NAT}$$
  5 $${DRP}_{\left(dir,mat\right)}^{*} = \frac{{(ERC}_{INT} {\text x} {DRP}_{INT}) - {(ERC}_{NAT} {\text x} {DRP}_{NAT})}{{ERC}^{*}}$$
  where ${ERC}^{*}$ is defined as the gain in information content from the international evaluation. For the direct effect, ${ERC}^{*}$ and its associated ${DRP}_{dir}^{*}$ was set to 0 if ${ERC}_{dir}^{*}$ was less than 0.2. The ${DRP}_{mat}^{*}$ was blended only if ${ERC}_{mat}^{*}$ was greater than 0.1.
5. *Single-step genomic evaluation*: Using the single-step multi-trait national genomic model [5], we implemented a multi-trait single-step genomic evaluation with the derived ${DRP}_{dir}^{*}$ and ${DRP}_{mat}^{*}$ as genetically correlated traits to the direct and maternal effects of 200-DW. The model involved the national single-step genomic evaluation with the two additional traits modelled with a 0.97 genetic correlation between the national phenotype and the computed ${DRP}^{*}$.

**Fig. 1:**
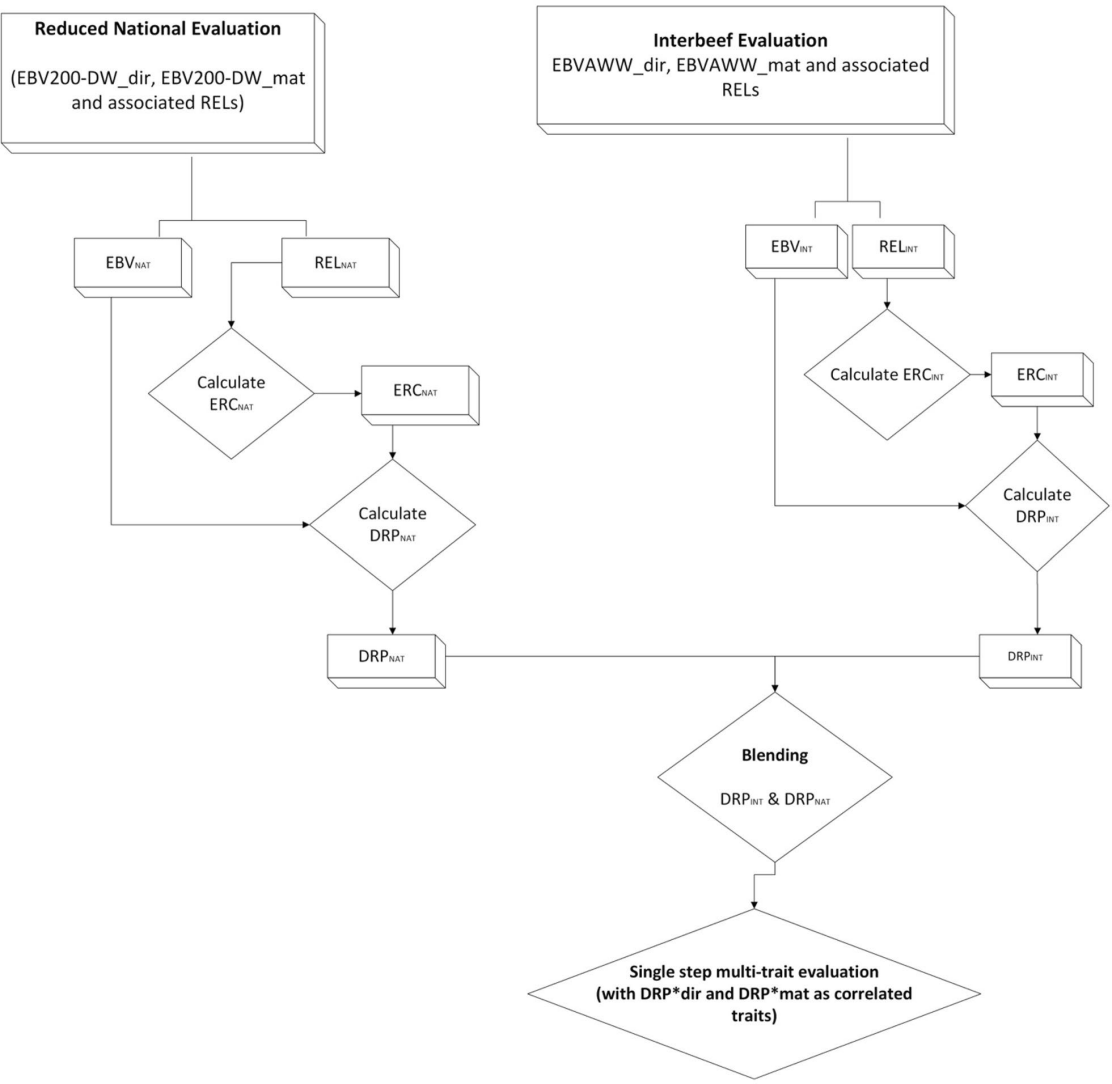
Workflow describing the integration of foreign EBVs into a National Single Step genomic evaluation system. EBV200-DW_dir: 200-day weight direct breeding value; EBV200-DW_mat: 200-day weight maternal breeding value; EBVAWW_dir: Interbeef Adjusted Weaning Weight direct EBV; EBVAWW_mat: Interbeef Adjusted Weaning Weight maternal EBV; EBV_NAT_: National EBV; REL_NAT_: National reliability; ERC_NAT_: National Effective Record contribution; DRP_NAT_: National deregressed proofs; EBV_INT_: Interbeef EBV (direct, maternal); REL_INT_: Interbeef reliability (direct, maternal); ERC_INT_: Interbeef Effective Record contribution (direct, maternal); DRP_INT_: Interbeef deregressed proofs (direct, maternal).

Considering that the direct and maternal EBVs are deregressed independently, steps two and three were carried out separately for direct and maternal genetic effects to obtain the ${ERC}_{IN{T}_{DIR}}$, ${DRP}_{IN{T}_{DIR}}$, ${ERC}_{IN{T}_{MAT}}$, and ${DRP}_{IN{T}_{MAT}}$ for the direct and maternal effects, respectively. This process was carried out using the routine Interbeef (AWW_dir_, AWW_mat_) and national (200-DW__dir_, 200-DW__mat_) traits. The workflow is described in Fig. 1.

All computations in this study were done using a combination of the MiX99 software suite and self-written code in Python and R. The matrix deregressions and reverse reliability calculations were carried out with MiX99 software [22], and the scalar deregressions were carried out using the code made available by [3] adjusted to our situation. The analysis and visualization were done using the regular data science suite of software available in Python.

### Evaluation

#### Reversibility test for the deregression of EBVs

The estimation of conventional EBVs can be understood as a process of regressing phenotype data on additive genetic effects using pedigree information. Deregression is a procedure that computes DRPs by reversing the regression process [13, 23, 29]. Three approaches were used to obtain DRPs. Deregression procedures can be generally categorized into iterative matrix [14] and noniterative scalar approaches [12, 13, 18]. We compared the DRPs obtained from the matrix approach to the scalar deregression approaches for all four breeds. The DRPs for the scalar approaches were calculated as described in step 3 above.

The reversibility of EBVs was evaluated using the correlation between the EBVs from phenotypes (*i.e. the EBVs deregressed to obtain the DRPs*) and the EBVs obtained from a single trait BLUP model with $y$ as the DRP and the calculated ERC as weights.

6 $$y= \mu + u+ \varepsilon$$

where y is the DRP for direct or maternal effects, $$\mu$$ is the general mean of the model, $$u$$ is the breeding value, and $$\varepsilon$$ is the residual effect with a normal distribution $$N\left( {0,{\raise0.7ex\hbox{${\sigma_{e}^{2} }$} \!\mathord{\left/ {\vphantom {{\sigma_{e}^{2} } {ERC }}}\right.\kern-0pt} \!\lower0.7ex\hbox{${ERC }$}}} \right)$$, where $${\sigma }_{e}^{2}$$ is the residual variance of the single-trait pedigree BLUP animal model with maternal effects.

For the computation of EBVs from the DRPs, we treat the direct and maternal effects as uncorrelated. For the direct EBVs, we considered only animals with a 200-DW phenotype in the national evaluation, and for the maternal traits, only animals that are dams of animals with a 200-DW phenotype.

#### GEBV comparison and validation

We used the Linear regression (LR) approach presented in [24] to evaluate the impact of integrating foreign data in the national evaluation. The LR method computes key indicators of accuracy, bias, and correlation between GEBVs estimated from a 'full' evaluation and a 'partial' evaluation where phenotypes from a focal group are masked. In this study, the focal group comprised animals born in the last three years (i.e., born after 2020). Considering the structure of German beef cattle breeding and the interest of breeders in blending foreign and national EBVs, a forward validation with production animals which broadly serve as selection candidates in the German beef cattle breeding system were used as focal animals in this study. The number of focal animals per breed is presented in Table 1. The LR validation was computed for the evaluation model incorporating foreign data (${GEBV}_{blended}$) and the national evaluation without foreign data (${GEBV}_{national}$). The regression coefficient (b1), with an expectation of 1.0, is defined as the regression coefficient of the GEBV obtained from the full evaluation on the GEBV obtained from the partial evaluation. Regression coefficients lower or larger than 1.0 indicate overdispersion or underdispersion of the (G)EBV, respectively. The Pearson coefficient between the GEBVs obtained from the full and partial evaluations is an estimator of the ratio between the accuracy of the partial and the accuracy of the full evaluation. We carried out four evaluations, two of which were full evaluations (*national*, *national* + *foreign*), where we used all available data. We carried out two additional evaluations with a partial dataset (*national*, *national* + *foreign*) where we masked the phenotypes of the focal group. We compared the GEBVs obtained from full and partial evaluations to assess the impact of foreign information and the deregression approach on the single-step genomic evaluation of four beef cattle breeds in Germany. Using the focal validation group, we benchmarked the evaluations with blended foreign information against a base national evaluation where no foreign information is blended.

**Table 1:** Number of animals across breeds

Breed	No. Phen	No. Ped	No. Genotypes	No. Interbeef EBVs	No. Validation animals
DA	142,809	199,126	5059	150,222	21,228
CHA	128,810	198,684	6162	137,504	13,687
SIM	144,338	222,789	5350	149,185	16,172
LIM	139,522	204,111	7754	148,419	17,222

DA: Deutsche Angus, CHA: Charolais, SIM: Simmental, LIM: Limousin. No. Phen: Number of animals with national phenotype (200-DW); No. Ped: Number of animals in the pedigree; No. Genotypes: Number of animals with genotype record. No. Interbeef EBVs: Number of animals with international EBV (AWW)

### Dataset

The phenotype, genotype, and pedigree data were obtained from the Vereinigte Informationssysteme Tierhaltung w.V (vit, Verden) (https://www.vit.de) database.

### Phenotypic data

The national phenotypic data were obtained from the routine official beef cattle evaluation of December 2022. The EBV_INT_ and associated reliabilities were obtained from the routine Interbeef evaluation of January 2023. Four German beef cattle populations participated in the Interbeef evaluations (Deutsche Angus (DA), Charolais (CHA), Limousin (LIM) and Simmental (SIM)). The Interbeef EBVs for AWW include the direct and maternal EBVs for German animals and international publishable sires already present in the German national pedigree on the German scale. The conditions for publishable sires are detailed in [2, 3]. A total of 585,330 Interbeef EBVs of German animals were available across the four breeds (DA: 150,222, CHA: 137,504, SIM: 149,185, LIM: 148,419). The total number of German animals (international publishable sires or animals in the German beef cattle pedigree) with Interbeef results is presented in Table 1.

### Pedigree and genotype data

Given that countries only have access to their national pedigrees, the pedigrees for the respective breeds were extracted from the German national pedigree database. The pedigree included beef cattle animals with phenotype records and their relatives. Table 1 presents the number of animals in the respective pedigrees and the number of genotyped animals. The genotypes of all animals in the breed-specific national pedigree with a genomic record were used in the evaluation. The animals were genotyped using Eurogenomics panels (EuroG MD V1.1, EuroG MD V2, EuroG MD V3, EuroG10K V7, and EuroG10K) and the Illumina 50 K V2 chip. Quality control of the SNP dataset applied a minor allele frequency (MAF) threshold of ≥ 0.01. The final dataset included 45,613 SNPs per animal.

## Results

The matrix deregression approach had an almost perfect correlation (> 0.99) between the EBVs obtained directly from the phenotype (EBV_i_) and the EBVs obtained from the DRPs (EBV_j_) (Table 2). Although, the Interbeef trait AWW and the national trait 200-DW are based on body weight measurements recorded around the weaning age of the animal, the EBVs are estimated differently. The 200-DW is typically measured between the 90th and 280th day of life and the age at measurement is adjusted as a regression coefficient in the MME during the estimation of EBVs. For AWW, the recorded weaning weight phenotype is linearly adjusted for the age of the animal at age of recording prior to sending the phenotype to the Interbeef evaluation centre. Furthermore, taking into consideration that the initial EBVs for AWW and 200-DW were estimated with a different pedigree basis, Table 2 shows the results on a separate basis for 200-DW and AWW. In the case of AWW where EBV_i_ was estimated from a larger Interbeef pedigree, and EBV_j_ estimated from DRPs derived from only the national pedigree (a subset of the Interbeef pedigree). This is a different scenario to the evaluation of 200-DW where both EBV_i_ and EBV_j_ were estimated from the national pedigree. The correlation between the EBVs obtained from the original phenotypes and the EBVs obtained from the pseudo-phenotypes ranged between 0.75 and 0.99 for the scalar deregression methods. Across breeds, the correlation between EBV_i_ and EBV_j_ was consistently less than the expected value of 1 for the scalar deregression approaches. In our evaluation of the scalar deregression approaches, the EBV_200-DW_dir_ showed the lowest correlation across the four breeds in our analysis. Within the scalar approaches, similar correlations between EBV_i_ and EBV_j_ were obtained for the Garrick and VanRaden deregression approaches in the maternal traits.

**Table 2:** The Pearson correlation and regression coefficient between the EBVs obtained from phenotypes and EBVs obtained from DRPs

Trait	Approach	Correlation				Regression coefficient			
		CHA	DA	SIM	LIM	CHA	DA	SIM	LIM
EBV_AWW_dir_	Garrick	0.90	0.90	0.89	0.91	0.71	0.71	0.66	0.73
EBV_AWW_mat_		0.94	0.94	0.94	0.94	0.84	0.82	0.87	0.84
EBV_200DW_dir_		0.75	0.77	0.82	0.88	0.60	0.70	0.78	0.92
EBV_200DW_mat_		0.98	0.99	0.99	0.95	0.97	0.99	1.01	0.92
EBV_AWW_dir_	Van Raden	0.93	0.93	0.93	0.94	1.13	1.13	1.12	1.11
EBV_AWW_mat_		0.94	0.94	0.94	0.94	1.29	1.24	1.27	1.28
EBV_200DW_dir_		0.90	0.90	0.90	0.91	1.07	1.09	1.06	1.09
EBV_200DW_mat_		0.98	0.98	0.98	0.98	1.15	1.13	1.13	1.14
EBV_AWW_dir_	Matrix	1.00	1.00	1.00	1.00	1.00	1.00	1.00	1.00
EBV_AWW_mat_		1.00	1.00	1.00	1.00	1.00	1.01	1.01	1.00
EBV_200DW_dir_		1.00	1.00	1.00	1.00	1.00	1.01	1.01	1.00
EBV_200DW_mat_		1.00	1.00	1.00	1.00	1.00	1.00	1.00	1.00

EBV_AWW_dir_: direct animal genetic effect of adjusted weaning weight; EBV_AWW_mat_: maternal genetic effect of adjusted weaning weight; EBV_200-DW_dir_: direct genetic effect of 200-day weight; EBV_200-DW_mat_: maternal genetic effect of 200-day weight

The results of the regression of the EBV obtained from DRPs on the EBV obtained from phenotype are also presented in Table 2. Generally, the Garrick's deregression approach showed overdispersion of EBVs in the national traits, especially in EBV_200DW_dir_, with the regression coefficients for EBV_200DW_dir_ ranging between 0.60 and 0.92. For the maternal effect (EBV_200DW_mat_) in the national evaluation, the regression coefficient ranged between 0.92 and 1.01. In the AWW evaluation, the Garrick deregression approach showed strong overdispersion of the direct effect (0.66–0.73). Similar to the 200-DW traits, the maternal effect (EBV_AWW_mat_) ranging between 0.82 and 0.87 showed less overdispersion compared to the direct effects. The Van Raden approach showed slight underdispersion, ranging between 1.06 and 1.09 in EBV_200DW_dir_. The regression coefficient of EBV_AWW_dir_ ranged between 1.11 and 1.13 across all breeds. The regression coefficients from the maternal effects ranged between 1.24 and 1.29 for EBV_AWW_mat_, and 1.13–1.15 for EBV_200DW_mat_. As expected, the matrix deregression approach also has an unbiased regression coefficient, ranging between 1.00 and 1.01. Across all breeds, traits, and methods, the standard errors for the regression coefficient presented in Table 2 were less than 0.004. In general, the results indicate that the Garrick approach showed overdispersion of the EBVs derived from the DRPs and the VanRaden deregression approaches tends to yield underdispersion in the EBVs derived from DRPs across all breeds and traits.

The correlation coefficient between EBV_i_ and EBV_j_ were similar in scalar deregression approaches, with the EBV_AWW_dir_ ranging between 0.89 and 0.91 in the Garrick deregression approach and the correlation coefficient in the Van Raden approach being slightly higher for EBV_AWW_dir_ ranging between 0.93 and 0.94 across the four breeds. The correlation coefficient was lowest in the Garrick deregression approach for EBV_200DW_dir_ ranging between 0.75 and 0.88 across the four breeds. For the trait EBV_200DW_dir_, the Van Raden deregression approach showed higher correlation between EBV_i_ and EBV_j_ ranging between 0.90 and 0.91. Similar to the result from the regression coefficient, the matrix deregression approach showed a perfect correlation between EBV_i_ and EBV_j_.

**Fig. 2:**
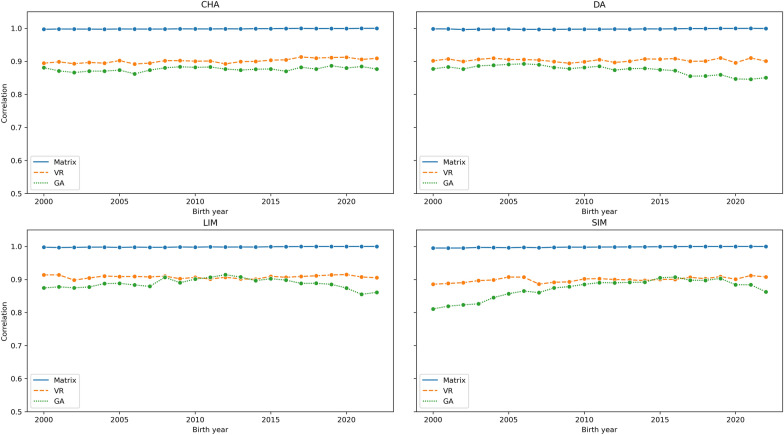
The correlation between the EBVs calculated from phenotype and the EBVs calculated from deregressed proofs (AWW_dir). CHA: Charolais, DA: Deutsche Angus, LIM: Limousin, SIM: Simmental, AWW_dir: Adjusted Weaning Weight (Direct EBV)

For the AWW_dir Interbeef trait, Fig. 2 shows the correlation between the EBVs obtained from the original phenotype and the EBVs obtained from pseudo-phenotypes for the four breeds in our analysis across birth years. The matrix approach shows a stable correlation across the birth years. The correlation in the Van Raden approach was consistently higher than the Garrick approach across all breeds.

**Fig. 3:**
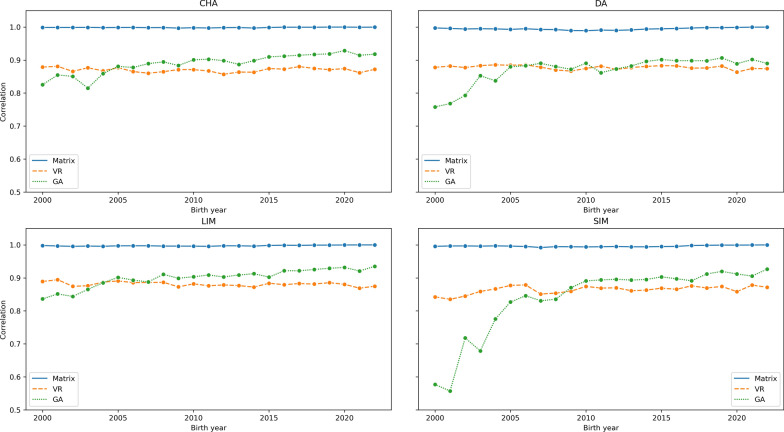
The correlation between the EBVs calculated from phenotype and the EBVs calculated from deregressed proofs (200-DW_dir). CHA: Charolais, DA: Deutsche Angus, LIM: Limousin, SIM: Simmental. Matrix: Matrix deregression approach, VR: Van Raden deregression approach, GA: Garrick deregression approach, 200-DW_dir: 200-day weight (Direct EBV).

Figure 3 shows the correlation between the pedigree-based EBVs calculated from phenotype and the EBVs calculated from DRPs across birth years for 200-DW_dir. The matrix approach shows a consistent correlation close to the expected value of 1 across all birth years and breeds.

Table 3 shows the regression coefficient (b1) of regressing the GEBVs obtained from a full evaluation on a truncated evaluation using only national data and the blended GEBVs across the three deregression approaches. For the traits in which we did not directly blend foreign EBVs (BW, 200-DM, 365-DW, 365-DM), the regression coefficients were similar between the purely national evaluation and the evaluations blended with foreign EBVs. The 200-DW trait, which was blended with foreign EBVs, showed moderate to slight over-dispersion, with the b1 ranging between 0.90 and 0.98 for the purely national evaluation. The Garrick deregression approach showed higher levels of over-dispersion across all breeds, particularly with the breed DA showing a significant over-dispersion with a regression coefficient of 0.65. The b1 values obtained from the evaluation where DRPs were obtained using the Van Raden (0.94–1.05), and matrix deregression (0.89 and 0.97) approach showed slight to almost no dispersion. Using the DRP obtained from the matrix deregression approach, the b1 coefficient ranged between 0.81 and 0.94 from the evaluation including foreign EBVs improved the over-dispersion shown in the purely national evaluation (b1: 0.81–0.85) for the maternal effect. The evaluations where DRPs from the scalar deregression approaches were blended performed at par with the base national evaluation with GA derived DRPs ranging between 0.61 to 0.82 and the evaluation with VR derived DRPs ranging between 0.82 and 0.86 for 200-DW_mat.

**Table 3:** The regression coefficient (b1) metrics for the validation animals

Traits	National				Nat + Foreign (Matrix)				Nat + Foreign (GA)				Nat + Foreign (VR)			
	CHA	LIM	SIM	DA	CHA	LIM	SIM	DA	CHA	LIM	SIM	DA	CHA	LIM	SIM	DA
BW	1.02	1.01	0.96	0.99	1.02	0.99	0.97	0.99	1.02	0.99	0.96	0.98	1.02	1.01	0.97	1.00
200-DW	0.95	0.98	0.90	0.96	0.97	0.89	0.95	0.97	0.93	0.89	0.86	0.65	0.99	1.01	0.94	1.05
365-DW	0.99	1.02	0.93	1.02	1.00	1.00	0.95	1.03	1.00	1.00	0.94	0.97	1.02	1.03	0.96	1.04
200-MS	1.03	0.98	0.98	0.95	1.04	0.95	1.00	0.96	1.02	0.95	0.93	0.82	1.05	0.99	0.97	0.99
365-MS	1.05	1.01	0.97	1.02	1.05	1.01	0.99	1.03	1.05	1.01	0.97	0.96	1.07	1.02	0.98	1.02
200DW_mat	0.81	0.83	0.81	0.85	0.92	0.81	0.94	0.92	0.82	0.81	0.81	0.61	0.86	0.85	0.85	0.82

BW**:** Birth Weight; 200-DW: 200-day weight (Direct effect); 356-DW: 365-day weight; 200-MS: 200-day muscle score; 200DW_mat: 200-day weight (Maternal effect); National: Evaluation with only national data; Nat + Foreign: Evaluation with national data blended with foreign data. SE ranged between 0.004 and 0.008 across all breeds and evaluations

Table 4 presents the ratio of accuracies between the EBV obtained in the partial evaluation and the full evaluation for the validation animals with phenotypes in the full evaluation. For 200-DW in the base national evaluation, the ratio of accuracies between the GEBVs obtained from the full and partial evaluations ranged between 0.76 and 0.78. For CHA, LIM, and SIM, there were no significant changes in the ratio of accuracies between full and truncated GEBVs in the base national evaluation and the GEBVs derived from evaluations blended with foreign EBVs using the matrix and GA approaches. Relative to the GEBVs derived from the base national evaluation, the GEBVs derived from the evaluations blended with foreign EBVs using the VR approach showed considerable increase in ratio of accuracies between the full and truncated evaluations. The ratio of accuracies in the evaluation that blended matrix-derived DRPs ranged between 0.77 and 0.79 in 200-DW. The evaluation blended with scalar DRPs ranged between 0.51 and 0.80 for the evaluation blended with DRPs derived from Garrick approach, and 0.82 to 0.84 for the evaluation where DRPs derived from VanRaden deregression approach. In the evaluations where DRPs derived from the Garrick approach, the ratio of accuracies between the full and truncated GEBVs were 0.51 for direct effects and 0.64 for maternal effects for DA. Relative to the base national evaluations (0.72–0.74), the evaluations in which matrix-derived DRPs were blended with the national single step evaluation, there was a marked increase in the ratio of accuracy ranging between 0.73 in LIM, and 0.87 in DA.

**Table 4:** Ratio of accuracies between GEBVs obtained from the truncated and full evaluations for the validation animals

Traits	National				Nat + Foreign (Matrix)				Nat + Foreign (GA)				Nat + Foreign (VR)			
	CHA	LIM	SIM	DA	CHA	LIM	SIM	DA	CHA	LIM	SIM	DA	CHA	LIM	SIM	DA
BW	0.84	0.79	0.76	0.76	0.84	0.79	0.76	0.76	0.84	0.79	0.77	0.76	0.85	0.80	0.77	0.77
200-DW	0.78	0.78	0.76	0.77	0.79	0.78	0.77	0.77	0.80	0.78	0.77	0.51	0.84	0.84	0.83	0.82
365-DW	0.83	0.84	0.83	0.84	0.84	0.85	0.84	0.84	0.85	0.85	0.85	0.79	0.86	0.86	0.86	0.86
200-MS	0.81	0.78	0.78	0.72	0.82	0.79	0.78	0.72	0.83	0.79	0.77	0.62	0.84	0.81	0.80	0.75
365-MS	0.85	0.83	0.84	0.79	0.85	0.85	0.85	0.80	0.86	0.85	0.85	0.76	0.87	0.86	0.86	0.81
200DW_mat	0.73	0.74	0.72	0.73	0.86	0.73	0.87	0.87	0.71	0.73	0.71	0.64	0.74	0.77	0.74	0.76

BW**:** Birth Weight; 200-DW: 200-day weight (Direct effect); 356-DW: 365-day weight; 200-MS: 200-day muscle score; 200DW_mat: 200-day weight (Maternal effect); National: Evaluation with only national data; Nat + Foreign: Evaluation with national data blended with foreign data

For the focal group, Table 5 presents the level bias (in genetic standard deviations) between the GEBVs obtained from the truncated and full evaluations. Consistent with the other LR metrics, there were no differences between the base national and the evaluations that blended foreign information for the traits where no foreign information was blended.

**Table 5:** Level bias (in genetic SD) between GEBVs obtained from the truncated and full evaluations for the validation animals

Traits	National				Nat + Foreign (Matrix)				Nat + Foreign (GA)				Nat + Foreign (VR)			
	CHA	LIM	SIM	DA	CHA	LIM	SIM	DA	CHA	LIM	SIM	DA	CHA	LIM	SIM	DA
BW	0.39	0.32	0.32	0.34	0.39	0.32	0.32	0.33	0.37	0.31	0.32	0.34	0.37	0.31	0.31	0.32
200-DW	0.38	0.34	0.37	0.33	0.36	0.32	0.34	0.31	0.33	0.30	0.33	0.47	0.30	0.26	0.28	0.28
365-DW	0.37	0.34	0.37	0.33	0.36	0.33	0.35	0.32	0.33	0.31	0.33	0.37	0.32	0.30	0.31	0.30
200-MS	0.32	0.29	0.30	0.31	0.31	0.29	0.28	0.30	0.30	0.28	0.28	0.35	0.29	0.26	0.27	0.29
365-MS	0.31	0.29	0.29	0.30	0.30	0.28	0.28	0.29	0.28	0.27	0.27	0.31	0.28	0.26	0.26	0.28
200DW_mat	0.19	0.17	0.20	0.17	0.15	0.13	0.15	0.13	0.22	0.19	0.22	0.24	0.20	0.17	0.20	0.18

BW**:** Birth Weight; 200-DW: 200-day weight (Direct effect); 356-DW: 365-day weight; 200-MS: 200-day muscle score; 200DW_mat: 200-day weight (Maternal effect); National: Evaluation with only national data; Nat + Foreign: Evaluation with national data blended with foreign data

For 200-DW, the evaluation blended with VR-derived DRPs averaged 0.28 across all breeds. This was slightly higher in the evaluation with GA-derived DRPs (0.36) performing on par with the base national evaluation (0.35) and evaluation blended with matrix derived DRPs (0.33). More specifically, for the trait 200-DW, the VR approach showed the lowest level bias, ranging between 0.26 and 0.30 across the four breeds in this study. This was lower than the GEBVs obtained using the GA approach (0.30–0.47), matrix (0.31–0.36), and base national model (0.33–0.38). For 200-DW_mat, the matrix approach showed the lowest level bias between GEBVs obtained from the full and truncated evaluations. The matrix approach (0.13–0.15) slightly outperforms the base national (0.17–0.20), VR approach (0.17–0.20) and GA approach (0.19–0.24).

## Discussion

With increasing globalization and the exchange of cattle semen, the integration of EBVs obtained from national and international evaluations will continue to enhance the genetic potential of livestock populations. The optimal blending of these EBVs allows for the accurate selection of a broader set of animals using all available sources of information [4]. The benefits of blending foreign information with national evaluations is particularly important in countries with limited national data [3]. This study validated deregression approaches to combine international Interbeef EBVs with a national single-step genomic evaluation. Here, we discuss the results obtained from the different steps of the blending approach. We examine iterative and scalar approaches to deregression and the reversibility of the derived DRPs. Furthermore, using the three deregression approaches studied, we discuss the impact of integrating foreign EBVs into the German national single-step evaluation in a forward validation study. We discuss these findings in the context of the four German Beef cattle populations participating in the official Interbeef evaluation.

### Reversibility test for the deregression of EBVs

Conventional breeding value estimation can be understood as regressing phenotype data on additive genetic effects using pedigree information. The deregression of EBV reverses the regression process to obtain the pseudo-phenotype data from EBVs [25]. Generally, for an unbiased deregression, the expected Pearson correlation between the EBVs obtained from phenotypes and EBVs obtained from DRPs is 1 [7]. Compared to scalar deregression approaches, the matrix deregression approach showed an almost perfect correlation to the initial EBV for all breeds (Table 2). This is in agreement with literature where the simultaneous matrix deregression outperforms scalar deregression methods [26, 27]. Our results agreed with [13] where, in comparison to the scalar deregression approaches, the EBVs obtained from matrix-based-DRPs had a correlation greater than 0.99 with the original EBVs obtained from phenotypes.

In a further analysis that split the correlation between EBV_i_ and EBV_j_ between birth years (Figs. 2 and 3), the matrix approach shows a consistent Pearson correlation > 0.99 across all traits, breeds, and birth years. The correlation coefficient of the scalar approaches showed slightly higher variability and was consistently lower across the birth years relative to the matrix approach. Particularly, the low correlation observed in the SIM, and to a lower extent in DA in animals born between 2000 and 2007 is likely due to pedigree incompleteness. The two breeds affected by the low correlation are peculiar in a similar manner. The DA population is a sub-population of the global Aberdeen Angus breed which is considered a separate breed by the German breeding organizations. The SIM breed is also split into the beef and dairy lines which are organized into different breeding societies. Given these sub-population structures and the incompleteness of the pedigrees, we theorize these are the cause of biases observed in EBVs as estimated from the scalar approaches.

### GEBV comparison

This study aimed to compare and validate deregression approaches in integrating international pedigree-based EBVs into single-step national evaluations of German beef cattle populations. To investigate this, we compared a single-step national evaluation with a single-step national evaluation that integrated foreign data. We carry out a national single step genomic evaluation with DRPs obtained from the three deregression approaches. The validation results from the focal group across the five national traits are presented in Tables 3, 4 and 5. The national breeding value model is a multi-trait model with five traits and six effects, with 200-DW having a direct and maternal effect. The national traits (EBV_200-DW_dir_ and EBV_200-DW_mat_) receive additional information (EBV_AWW_dir_ and EBV_AWW_mat_) from the Interbeef evaluation. For the other national traits without foreign information (BW, 200-DM, 365-DW, 365-DM), we expected only a slight change between the evaluations from the genetic covariance between traits that receive foreign data and other traits in the multi-trait evaluation.

Overall, the results from our study show that blending foreign information with the matrix deregression approach in the national single-step genomic evaluation performs at par with (200-DW) or improves on (200-DW_mat) the base national evaluation, which does not include foreign information for focal group. Our results agree with the assertions by Bonifazi [28] who argued that integration of foreign information reduces level bias and results in similar dispersion. Table 3 shows the regression coefficient from regressing the full on truncated GEBVs for the three deregression approaches. For the blended traits (200-DW and 200-DW_mat), we find that the regression coefficient was closer to 1 in the evaluation using the matrix deregression approach. Compared to the base national model, integrating foreign information using a matrix deregression approach improved the regression coefficient for the direct and maternal GEBVs. This is consistent with literature where improved dispersion of GEBVs is observed in evaluations integrated with foreign information relative to national evaluations [3]. For the direct genetic effect (200-DW) the evaluation with the DRPs obtained using the GA approach showed more overestimation across all breeds. Integrating foreign information using the VR deregression approach showed regression values close to 1, even slightly outperforming the base national and the matrix-based evaluation. For the 200-DW_mat GEBVs, the base national evaluation showed significant over-dispersion with regression coefficient ranging between 0.81 and 0.85. Further supporting the results obtained by Bonifazi [28], the three approaches to blending foreign EBVs with the national evaluation showed improvements relative to the base national model.

In evaluating the ratio of accuracies between the GEBVs obtained from the full and partial evaluations (Table 4), the evaluation in which the GEBVs were blended using foreign data with the VR approach outperformed the base national model, GA approach, and the matrix approach. The matrix approach performed on a similar level to the base national evaluation. For the 200-DW_mat GEBVs, the evaluation in which we blended foreign information using the matrix approach outperformed the base national evaluation and the scalar-based blending approaches.

For the level bias of GEBVs obtained from full and truncated evaluations (Table 5), the evaluations that blended foreign information showed a lower level bias between the GEBVs obtained in the truncated and full evaluations. For the 200-DW, the base national model had a level bias ranging between 0.33 and 0.38. All three approaches to integrating foreign information had lower level bias between the GEBVs obtained from the truncated and full evaluation for the focal group. For the 200-DW effect, the VR approach had a slightly lower level bias in comparison to the matrix and GA. For 200-DW_mat, the matrix approach outperformed the base national, GA, and VR approaches. Overall, we found the GA approach to deregressing and blending foreign information performed the worst, particularly for the breed DA in this study. Furthermore, the result from this study indicate that the VR approach performs on par with the matrix approach in the integration of foreign information for 200-DW. However, for the 200-DW_mat, the matrix evaluation outperformed the scalar and base national evaluations. Considering that we only had access to the German national pedigree, the calculation of ERC for the Interbeef traits was likely underestimated due to the absence of the information of progenies that may not be available in the German national pedigree. A more accurate estimation of ERCs, ${ERC}^{*}$, and subsequently DRP*, would enhance the effective utilization of foreign data at the national level. One potential service improvement from Interbeef could involve providing DRPs and ERCs, alongside EBVs, to the participating countries. While the matrix approach yielded the most consistent results across all breeds, its application in calculating ERCs and DRPs may depend on the availability of matrix-based deregression methods for approximating EBV and REL into DRP and ERC within certain commercial software packages, either at the national level or within Interbeef [3, 11, 28]. Our study indicates that the VR deregression approach could be a viable alternative for calculating DRP* and integrating foreign data into national evaluations, thereby enabling a more precise blending of international EBVs with national evaluations, whether conventional or single-step.

## Conclusions

This study validates approaches for integrating foreign, Interbeef direct and maternal effect estimates into the national single-step evaluations of four German beef cattle breeds. Our analysis revealed that the matrix deregression approach outperforms scalar approaches, generally exhibiting a higher correlation with the original EBVs across the various breeds and traits in our study. This study’s central focus on integrating foreign data into the German national single-step evaluation culminates in a comparison of GEBVs obtained from a base national single-step genomic evaluation and three single-step genomic evaluations blended with international information. Utilizing the scalar deregression approaches, particularly the VR deregression approach, we found improvements in regression coefficients and correlations, particularly for 200-DW over the base national evaluation. However, the matrix deregression approach performed at par or better than the base national evaluation and the scalar-based deregression methods for the traits and breeds in this study.

## Data Availability

The data supporting this study’s findings may be available upon request from vit Verden. However, restrictions apply to the availability of these data, which were used under a licence of a material transfer agreement for the current study and thus are not publicly available.
